# An update on tests used for intraoperative monitoring of cognition during awake craniotomy

**DOI:** 10.1007/s00701-024-06062-6

**Published:** 2024-05-07

**Authors:** Beleke de Zwart, Carla Ruis

**Affiliations:** 1 Experimental Psychology, Helmholtz Institution, Utrecht University, Utrecht, The Netherlands; 2 Department of Neurology and Neurosurgery, University Medical Center Utrecht, Utrecht, The Netherlands

**Keywords:** Cognition, Neuropsychological tests, Intraoperative monitoring, Brain neoplasms, Glioma, Epilepsy

## Abstract

**Purpose:**

Mapping higher-order cognitive functions during awake brain surgery is important for cognitive preservation which is related to postoperative quality of life. A systematic review from 2018 about neuropsychological tests used during awake craniotomy made clear that until 2017 language was most often monitored and that the other cognitive domains were underexposed (Ruis, J Clin Exp Neuropsychol 40(10):1081–1104, 218). The field of awake craniotomy and cognitive monitoring is however developing rapidly. The aim of the current review is therefore, to investigate whether there is a change in the field towards incorporation of new tests and more complete mapping of (higher-order) cognitive functions.

**Methods:**

We replicated the systematic search of the study from 2018 in PubMed and Embase from February 2017 to November 2023, yielding 5130 potentially relevant articles. We used the artificial machine learning tool ASReview for screening and included 272 papers that gave a detailed description of the neuropsychological tests used during awake craniotomy.

**Results:**

Comparable to the previous study of 2018, the majority of studies (90.4%) reported tests for assessing language functions (Ruis, J Clin Exp Neuropsychol 40(10):1081–1104, 218). Nevertheless, an increasing number of studies now also describe tests for monitoring visuospatial functions, social cognition, and executive functions.

**Conclusions:**

Language remains the most extensively tested cognitive domain. However, a broader range of tests are now implemented during awake craniotomy and there are (new developed) tests which received more attention. The rapid development in the field is reflected in the included studies in this review. Nevertheless, for some cognitive domains (e.g., executive functions and memory), there is still a need for developing tests that can be used during awake surgery.

**Supplementary Information:**

The online version contains supplementary material available at 10.1007/s00701-024-06062-6.

## Introduction

Awake craniotomy is currently the growing standard for the majority of newly diagnosed gliomas and remains an essential technique in epilepsy surgery in crucial functional areas [258, 265]. Aside from increased tumor resection and optimal seizure control, awake brain surgery is related to more neurological and cognitive preservation [56, 84]. Historically, during awake brain surgery, language and motor function were most often mapped [52, 258]. However, the scope of neurocognitive deficits associated with gliomas and epilepsy extend far beyond the language and motor domains to, i.e., visuospatial, planning, attention, and social cognition [91, 178]. According to previous work, the bigger extent of domains that is monitored, the larger and safer the resections and the more cognitive functions will be preserved [63]. Besides, due to the prolongation of life expectancy after glioma resection, there is a need of maintaining a similar quality of life after surgery as before surgery [64, 84]. Therefore, next to mapping language and motor functions, attention is currently directed towards monitoring and sparing of neural networks that subserve (higher-order) cognitive processes. For example, executive functions are highly related to quality of life in glioma patients after awake brain surgery. Whereas sometimes going unnoticed in the hospital, serious problems can be experienced regarding planning or multitasking when returning to work [173, 204]. A recent review has outlined several other cognitive deficits that can have repercussions on a patient’s quality of life [64]. For instance, bimanual coordination is particularly important for individuals with musical and sport ambitions and conscious awareness is related to creativity and thus of high importance for artists [64]. What is more, proprioceptive deficits are related to problems in movement control and lowered independence in basic daily life activities [203]. Lastly, social cognition (e.g., mentalizing) is especially important in social interactions, and preservation of these functions is therefore necessary to prevent challenges in social behavior [176]. These examples demonstrate the high importance of extensive cognitive monitoring during surgery. This increased focus on enhancing the quality of life after surgery reflects the shift away from the traditional patient-centeredness, which aims to preserve a *functional* life for the patient, towards a more person-centered approach that prioritizes preserving a *meaningful* life for the patient [92].

Therefore, the aim of this study is to investigate whether this preferred change towards more differentiated mapping of cognitive functions has translated to a more varied set of tests used during awake surgical procedures. This study builds upon previous work that offered an overview of the neuropsychological tests used up until 2017 in patients suffering from brain tumors or epilepsy who underwent awake brain surgery [221]. The main conclusion held that language was indeed extensively monitored but that other cognitive domains received much less attention during awake brain surgeries and that there was a need of development of new tests. Since this systematic study was based upon included literature up to February 2017, we aim to build upon this work to investigate whether, and if so, what changes have taken place in the tests used for monitoring cognition by replicating the search with February 2017–November 2023 as incorporated time window [221]. Providing a new overview of the administered tests used during awake brain surgeries and comparing this with the results of the study of 2018 makes it possible to reveal recent developments in the field.

## Material and methods

A systematic literature search was conducted using PubMed and Embase from February 2017 up to November 2023 according to PRISMA (Preferred Reporting Items for Systematic Reviews and Meta-Analysis) guidelines [155]. We replicated the previously used framework to search our databases in which we combined diseases with awake brain surgery (disease [e.g., brain tumor, glioblastoma] AND procedure [e.g., craniotomy]) AND awake [e.g., monitoring, intraoperative]) [221]. For detailed search strategies per database, see the Supplementary.

**Fig. 1:**
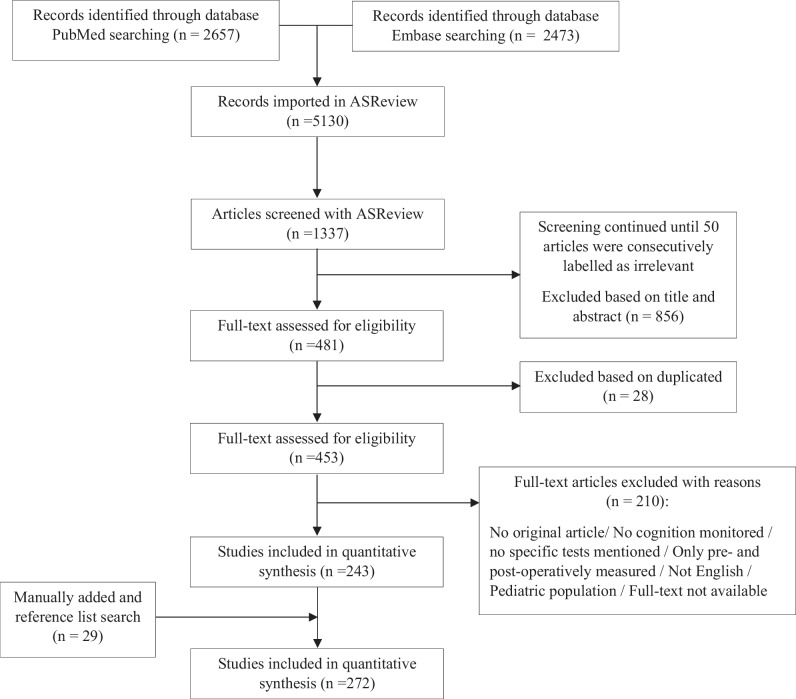
Preferred Reporting Items for Systematic Reviews and Meta-Analyses (PRISMA) flow diagram of the systematic literature search with the use of ASReview

Given that this systematic review builds upon the prior work, we employed the same approach with regard to in- and exclusion criteria (Fig. 1) [221]. We first screened the papers on title and abstract in which papers were excluded if the population was pediatric, when the language of the paper was other than English, when it was no original article (e.g., review, letter to the editor), when cognition was not monitored, or when the procedure did not comprise awake brain surgery [221]. Moreover, during the full-text assessment, a specific inclusion criteria was a clear description of the test or test paradigm used during surgery. This is especially relevant in the context of the sensory, motor, and somatosensory areas, since the procedure oftentimes starts with mapping these areas [259]. These domains were solely included when extensively studied by means of standardized tests instead of only reporting lack of sensations, movements, or control [221].

The machine learning algorithm ASReview is an artificial intelligence (AI) software and was utilized for screening the articles [238]. The software uses state-of-the-art active learning techniques to accelerate screening abstracts and titles by ranking literature on their textual proximity to previously relevant articles and is designed according to the principles of Open Source science. At first, all the articles that are derived from the database search are uploaded to the software. Beforehand, the researcher classifies minimal three articles as relevant to offer the tool a starting point. For every presented article after that, the researcher will label it as either relevant (inclusion) or irrelevant (exclusion). Based on this input, the software will first present articles which are textual close to the ones that are labeled as relevant. Since the software ranks the papers based on textual proximity, the chance that ASReview will present a relevant article diminishes with every consecutive *excluded* article. Therefore, our cutoff for stopping to scan title and abstract was set at 50 papers consecutively excluded, with the expectation that no relevant articles would be presented afterwards. For more details about the use of ASReview, we refer the reader elsewhere [238].

The database search yielded 5130 results (Fig. 1). The author screened by means of the active artificial machine learning tool ASReview 1337 articles for relevance based on title and abstract. In other words, after 1337 articles, we had labeled 50 papers *consecutively* as irrelevant and we stopped the screening process. This means that 3793 papers (5130 in total, minus 1337 that were screened) were not presented to us by ASReview, but these are with a high probability irrelevant. Out of these 1337 screened articles, 856 were excluded based on title and abstract. After removing duplicates, the 453 potentially relevant papers were assessed in full text for eligibility, resulting in a total of 243 included papers. Reference list search was applied and we added papers based on expert consultation, resulting in 272 included articles in total. Once the papers were selected from the literature databases, a description of the cognitive domains monitored during awake brain surgery and the tests or neuropsychological paradigms that were used were extracted from each included paper.

## Results

**Fig. 2:**
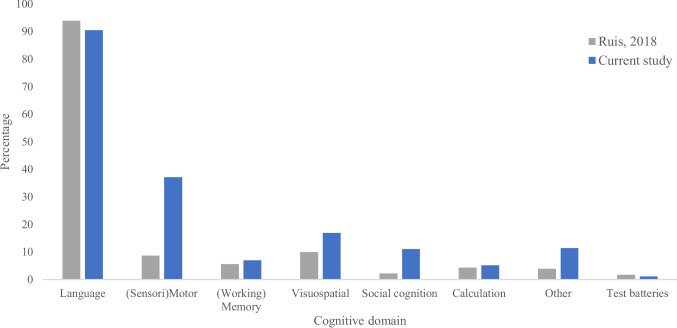
Percentages of studies reporting tests or paradigms per cognitive domain during awake brain surgery. *“Other” includes executive functions, clock reading, processing speed, left–right orientation, face recognition, musical skills, and proprioception

An overview of the final 272 included studies in this quantitative synthesis is presented in Table 1, with each cognitive domain and used test outlined per article. Standardized neuropsychological tests are presented in italic. Figure 2 presents a comparison of the percentual cognitive domains that were monitored, in order to compare our results with the reported domains in the work of 2018 [221]. As visible, the vast majority (90.4%) of included studies tested the language domain (Fig. 2). In 40% of these studies, *only* the language domain was tested, compared to 68% in previous work. Compared to 2018, there seems to be a trend towards more implementation of motor, visuospatial, emotion, and “other” tasks [221]. Because the “other” category has increased compared to the previous study, the cognitive domains and tests are described in more detail in Table 2. Within this category, proprioception, clock reading, left–right orientation, and processing speed are newly described cognitive domains. When interpreting the results, it should be taken into account that we did not statistically analyze the results.

**Table 1:** Studies included in the review, with cognitive domains monitored during surgery and test/paradigm used to assess the domain

Article	Cognitive domain	Description of test/paradigm
[10]	Language	Counting Reading (Quick Aphasia Battery) Picture naming (Quick Aphasia Battery)
[11]	Language	Counting Speak letters of alphabets Object naming Reading
	Motor	Performing simple movements
[12]	Language	Memory related queries – asking for the phone number, names of her dog
	Motor	Squeezing a squeaky toy
[13]	Language	Word comprehension (describing target word)
[14]	Motor + Higher order function	Counting + rhythmic contraction of the contralateral limb Calculation + rhythmic contraction of the contralateral limb
[15]	Language	Picture naming Semantic association task Reading aloud
	Visuospatial	Line bisection test Visual field test
	Social cognition	*Reading the Mind in the Eyes Test*
[16]	Language	Object picture-naming (*DO80*) Nonverbal semantic association test (*PPTT*)
	Social cognition	*Reading the Mind in the Eyes Test*
[17]	Language	Maintaining verbal contact
[18]	Language	Picture naming
[19]	Language	Counting
	Executive function	Switch counting and listing the alphabet (1-A-2-B)
[20]	Language	Picture naming Nonverbal semantic association test (*PPTT*)
	Motor	Repetitive movement upper limb
[21]	Language	Counting Alphabet recitation Visual naming Reading Token test
[22]	Language	Object naming Counting Conversation
[23]	Language	Object picture-naming (*DO80*)
[24]	Language	Counting Word generation test Naming
[25]	Language	Counting Picture object naming Nonverbal semantic association test (*PPTT*)
	Motor	Continuous repetitive movement of left superior limb
	Social cognition	*Reading the Mind in the Eyes Test*
[27]	Language	Naming task Spontaneous speech
[28]	Language	Singing
	Music	Tone and rhythm tasks
[29]	Language	Naming Reading
	Visuospatial	Time-to-contact test
[31]	Language	Counting Naming weekdays and months Picture naming Word repetition Sentence repetition Answering sentences and questions Spontaneous speech
[34]	Language	Image naming Pseudowords Verb generation
[35]	Language	Counting Picture naming Voluntary speech Auditory comprehension Reading Writing
	Motor	Continuous opening and closing of the hand
[36]	Language	Picture naming
	Social cognition	*Reading the Mind in the Eyes Test*
	Executive function	Trail Making Task part B
	Visuospatial	Line bisection task
[37]	Language	Picture-naming task Nonverbal semantic association (*PPTT*)
[38]	Language	Naming objects Naming animals Reading and answering written questions Participating in unstructured conversation
	Face recognition	Famous face naming
[39]	Language	Counting Naming task
	Motor	Making voluntary movements
[40]	Language	Picture naming Nonverbal semantic association test (*PPTT*)
	Language + Motor	Counting simultaneously to repetitive movement of right superior limb
[41]	Language	Object naming
[42]	Language + Motor	Naming simultaneously to contralateral upper extremity motor tasks
[43]	Language	Free dialogue Counting Naming days of the week, months Picture Object naming Action naming
[44]	Language	Object naming
	Language + Motor	Object naming + moving contralateral arm
	Motor	Continuous movements ( Knitting, Playing a musical instrument, Assembling auto parts)
[45]	Language	Object-naming task
[46]	Language	Counting Picture naming test Nonverbal semantic association test (*PPTT*)
	Motor	Repetitive movement of the right arm
	Language + Motor	Repetitive movement of the right arm + counting, picture naming test, nonverbal semantic association test
[47]	Language	Counting Object picture-naming (*DO80*) Nonverbal semantic association (*PPTT*)
[48]	Language	Counting Visual object naming task
	Motor	Continuous motor function
[49]	Language	Counting Reading (from a PowerPoint)
[50]	Language	Picture naming Sentence planning: describe the spatial relation between geometric shapes (E.g., the blue triangle is above the red circle)
	Motor	Limb movements
[51]	Language	Sentence repetition
	Motor	Moving and feeling through the right upper and lower extremities
[53]	Language	Naming Semantic Association Task
	Praxis	Hand Manipulation Task (*HMt*)
	Visuospatial	Double Pictures Naming Task (*DPNT*) Intraoperative Visual Task (*iVT*)
[54]	Language	Object-picture naming (*DO80*) Nonverbal semantic association (*PPTT*)
	Language + Motor	Object naming + continuous limb movement
[55]	Language	Counting Object-picture naming (*DO80*)
[57]	Language	Counting Picture naming task (*DO80*) Picture naming task with Virtual Reality Headset Reading Complex word repetition Nonverbal semantic association test (*PPTT*) Spontaneous speech production
[58]	Language	Object naming (Spanish)
[59] Video	Language	Word repetition Naming
[60]	Language	Word repetition Object naming Spontaneous speech Sentence completion Semantic odd-picture-out Semantic judgment (belong two target words to the same semantic category? And if so, what category?) Semantic judgment (belongs target word to a specific category?)
[61]	Multidomain testing (test battery)	RTNT
	Music	Listening to music
[62]	Language	Counting Free dialogue Stroop test used to assess language function
	Motor	9-peg hole test Hand-arm-leg movement
[65]	Language	Counting
	Language + Motor	Naming + contralateral upper limb movement
	Visuospatial	Modified picture naming with 2 pictures placed diagonally on the screen for visual field monitoring
[66]	Language	Nonverbal semantic association (*PPTT)*
	Language + Motor	Continuous movement + naming
	Social cognition	*Reading the Mind in the Eyes Test*
	Visuospatial	Line bisection task Modified picture naming with 2 pictures placed diagonally on the screen for visual field monitoring
[67]	Music	Keyboard playing
	Motor	Finger tapping task
[68]	Language	Counting
	Executive function	Switch counting and listing the alphabet (1-A-2-B)
[69]	Language	Picture-naming
	Language + Motor	Repetitive right upper limb movement + language tasks
	Social cognition	*Reading the Mind in the Eyes Test*
	Visuospatial	Line bisection task Name 2 pictures placed diagonally in each opposite visual field
[70]	Language	Counting Object picture naming Reading Repetition Semantics
	Motor	Opening and closing hand Regular movement of the foot
	Calculation	Calculation
[71]	Language	Counting Object-picture naming (*DO80*)
[72]	Language	Object picture naming Counting
	Motor	Voluntary movement
[73]	Language	Counting Naming test
[74]	Language	Counting Naming
	Praxis	Hand Manipulation task (*HMt*)
	Praxis + Language	HMt + Verbal motor-monitoring
[75]	Language	Naming
	Praxis	Hand Manipulation task (*HMt*)
[76]	Language + Motor	Picture-naming task + Contralateral arm movement
	Praxis	Hand Manipulation task (*HMt*)
	Visuospatial	Visual field task
[77]	Language	Picture naming Naming to definition
[78]	Language	Naming Comprehension Repetition comprehension
	Motor	Simple motor functions
[79]	Language	Picture naming task
[80]	Language	Free talk Simple questions Recitation
[81]	Language	Naming task Repetition task Nonverbal semantic association (*PPTT)* Semantic pairs task *(SPT)*
	Motor	Drum playing
[83]	Language	Naming task (*BNT*) Word repetition Continuous speech
[84]	Language	Object naming *(BNT or DuLIP*) Spontaneous speech Counting Sentence repetition
	Motor	Finger tapping task
	Calculation	Calculation
[85]	Language	Counting Object naming Word repetition Sentence comprehension Spontaneous speech
[86]	Language	Object-picture naming (*DO80*)
[87]	Language	Counting Picture naming
	Executive function	*Stroop task*
[88]	Language	Object naming Verb Generation Comprehension + Semantic retrieval (e.g., A yellow sour fruit)
[89]	Language	Naming task Counting Naming days and months
[90]	Multidomain testing (test battery)	RTNT
[93]	Language	Object naming Auditory description naming Semantic task (Indicate if target has features of a specific category) Phonological task (Indicate whether pictured object starts with particular sound)
[94]	Language	Counting Picture naming Reading Listening comprehension Semantic association and judgment Writing
[95] Video	Language	Counting Picture naming Word generation
[96]	Language	Counting Picture naming Semantic association tasks
[97]	Language	Counting Naming
	Calculation	Calculation task
[100]	Language	Object picture naming (*DO80*) Nonverbal semantic association test (*PPTT*) Reading words and pseudowords
	Language + Motor	Counting + contralateral arm movement
[101]	Language	Counting Object-naming task (*DO80*)
	Language + Motor	Picture naming + contralateral arm movement
	Visuospatial	Line bisection task
[102]	Language	Counting Visual naming Auditory comprehension
[103]	Visuospatial	Object naming in opposite quadrants
[104]	Language	Picture naming (*DuLIP*) Spoken object naming (*ECCO*) Reading Spelling
[105]	Language	Picture-naming tasks Reading Writing Sentence Repetition
[106]	Language	Object naming
[107]	Language	Object naming
[108]	Language	Naming tasks Verb generation tasks
[109]	Multidomain testing (test battery)	Various functions, language and nonlanguage task (orientation, memory, and attention, automatic series, fluency, naming, repetition, reading, comprehension) (RTNT)
[110]	Motor	Hand-grasping task
[111]	Language	Counting (English and Hindi) Naming task (English and Hindi)
	Motor	Continuous flexion of the elbow Finger grasping task
	Social cognition	*Reading the Mind in the Eyes Test*
[112]	Language	Picture naming Spontaneous speech
[113]	Language	Counting Object naming task Verb naming tasks
[114]	Language	Word listening Japanese story-listening
[115]	Language	Picture naming Common noun naming Proper noun naming
	Face recognition	Famous face naming
[116]	Language	Counting Picture naming
[117]	Language	Reading Free dialogue
[119]	Language	Object naming of pictures and sign language
[120]	Language	Naming task (*Snodgrass and Vanderwart*)
[121]	Language	Counting Naming Reading
[122]	Language	Counting Naming
[123]	Language	Picture naming Single word reading Short-phrase sentence completion
[124]	Language	Structured word-production task (functional morpheme production)
[125]	Language	Object-naming (*DO80*)
[126]	(Sign) Language	Counting Picture-naming task Lexical decision task sign language (indicate if a sign was real or pseudo)
[127]	Language	Picture-naming task Nonverbal visual semantic decision task
	Motor	Opening and closing of the mouth Finger tapping task
	Language + Motor	Picture-naming + Flexion and extension contralateral arm Nonverbal semantic decision test + Flexion and extension contralateral arm
[128]	Language	Number counting Object naming task
[129]	Language	Counting Reciting days of the week/days/year Auditory responsive naming task
[130]	Language	Counting
[131]	Language	Counting Object-picture naming (*DO80*)
	Language + Motor	Picture naming + Right limb movement
[132]	Visuospatial	Line bisection task
[133]	Language	Picture naming Nonverbal semantic association (*PPTT*) Reading
	Visuospatial	Line bisection task Object naming in opposite quadrants
	Calculation	Calculation
	Social cognition	*Reading the Mind in the Eyes Test*
	Working memory	Spatial 2-back test
[134]	Language	Counting Naming items
	Language + Motor	Naming + Moving the right upper limb
[135]	Language + Motor	Counting + Motor task Picture object-naming (*DO80*) + Motor Nonverbal semantic association test (*PPTT*) + Motor
[136]	Language	Singing
	Motor	Guitar playing
[137]	Language	Picture naming Auditory comprehension Repetition of short sentences
	Memory	Recognition memory
[138]	Motor	Following verbal commands
[139]	Language	Counting Reading Semantic decision
[140]	Language	Object-picture naming (*DO80*) Nonverbal semantic association (*PPTT*)
	Social cognition	Emotion recognition (Pictures of Facial Affect)
[142]	Language	Nonverbal semantic association test (*PPTT*)
	Visuospatial	Line bisection task
[145]	Language	Object-picture naming (*DO80*) Nonverbal semantic association (*PPTT*)
	Language + Motor	Continuous movement upper limb + naming
[146]	Language	Object-picture naming (*DO80*) Nonverbal semantic association test (*PPTT*)
	Language + Motor	Object-picture naming (*DO80*) + Continuous repetitive movement of superior limb
	Social cognition	Emotion recognition
[147]	Language	Nonverbal semantic association test (*PPTT*)
	Executive function	Trail Making Test B (*TMT-B*; tablet)
	Visuospatial	Line bisection task (tablet)
[148]	Language	Picture naming Verb generation Nonword repetition Speech articulatory agility maneuvers
[149]	Language	Counting Object picture-naming task (*DO80*) Reading task
	Memory	N-back memory task Recalling of pictures
[150]	Language	Counting Object-picture naming (*DO80*)
[151]	Language	Counting Picture naming tasks
	Calculating	Calculation task
[152]	Language	Picture naming
[153]	Language	Counting Naming Word-generation task
[154]	Language	Object naming Short question answers (with and without voice production)
	Motor	Finger tapping task Foot movement
[156]	Language	Conversation
[157]	Language	Object picture-naming task (*DO80*) Nonverbal semantic association test (*PPTT*)
	Social cognition	*Reading the Mind in the Eyes Test*
[158]	Motor	Voluntary movements
[159]	Language	Picture-naming
	Working memory	Digit span test Visual N-back task
	Visuospatial	Line bisection task
[160]	Language	Counting Picture-naming
	Working memory	Digit span Visual N-back task
	Visuospatial	Line bisection task
[161]	Language	Counting tasks Picture-naming tasks
	Working memory	Visual N-back test Digit span
	Motor	Movement of the upper and lower limb
	Visuospatial	Line bisection task
[162]	Language	Counting Picture naming
[163]	Language	Object naming Counting
[164]	Language	Picture-naming
[165]	Language	Counting Picture naming tasks
	Social cognition	Emotional sensitivity task
[166]	Language	Object naming Fluency
[167]	Language	Counting
	Working memory	Spatial-to-back test
	Visuospatial	Line bisection task
[168]	Language	Naming Reading Auditory comprehension Repetition Free conversation
[169]	Language	Answer short auditory questions (e.g., what flies in the sky) Syllable repetition Counting Reciting ABC’s Humming
[170]	Visuospatial	Line bisection task
[171]	Motor	Simultaneously move finger and elbow
[172]	Social cognition	Emotion recognition based on eyes
	Visuospatial	No specific test mentioned
	Working memory	No specific test mentioned
[174]	Language	Picture naming
	Visuospatial	Line bisection task
	Social cognition	False beliefs test Emotion recognition task
[175]	Language	Picture naming Word production
	Motor	Movement of an upper extremity
	Working memory	2-back test
	Visuospatial	Line bisection task
	Social cognition	Emotion recognition task Predicting others mental state
[176]	Language	Picture naming Nonverbal semantic association test (*PPTT*)
	Social cognition	*Reading the Mind in the Eyes Test*
	Visuospatial	Line bisection task
[177]	Language	Object naming (290 drawings) Reading Distinguishing words from pseudowords Semantic decision making (same category y/n?)
	Language + Motor	Verbal tasks + Motor movements
	Motor	Opening + closing hand Flexion + tension foot Complex movement such as screwing a nut
	Visuospatial	Line bisection task Object naming in opposite quadrants
	Social cognition	Emotion recognition task
	Executive function	Go/No-go task
	Working Memory	Memorize stimuli, then distraction: is this stimuli the same as previous or not?
	Calculation	Calculation
[178]	Language	Picture naming Nonverbal semantic association test (*PPTT*)
	Social cognition	*Reading the Mind in the Eyes Test*
[179]	Language	Counting Object picture-naming task (*DO80*) Semantic association task
	Language + Motor	Counting/*DO80* + Contralateral movement
[180]	Language	Number counting Picture naming task Verbal semantic association task *(PPTT)*
	Motor	Upper limb movements
	Visuospatial	Line bisection task
[181]	Language	Counting Picture naming
[182]	Language	Counting
	Motor	Finger grasping Sticking out tongue Moving the fingers
[183]	Language	Naming items
	Motor	Finger tapping task
[184]	Language	Picture naming Word reading Spontaneous speech
[185]	Language	Free conversation Picture naming Responsive naming
[186]	Language	Object naming
[187]	Language	Counting Object-picture naming (*DO80*)
[188]	Language	Counting Object-picture naming (*DO80*)
	Motor	Continuous flexion and extension of the upper limb
[189]	Language	Object-picture naming (*DO80*) Nonverbal semantic association (*PPTT*)
	Social cognition	Modified version *Reading the Mind in the Eyes Test*
[190]	Language	Counting
	Working memory	Digit span
	Visuospatial	Visual symbol recognition task
[192]	Language	Counting Visual naming
[193]	Language	Counting Recite the alphabet
[194]	Language	Nonverbal semantic association test (*PPTT*)
	Calculation	Calculation
	Social cognition	*Reading the Mind in the Eyes Test*
[196]	Language	Picture naming task
	Music	Playing the violin
[197]	Language	Counting Object picture-naming (*DO80*)
[199]	Language	Number counting (1-10), Object picture-naming (*DO80*) Nonverbal semantic association test (*PPTT*)
	Language + Motor	Naming + left arm movement
	Face recognition	Famous face naming
	Social cognition	*Reading the Mind in the Eyes Test*
	Visuospatial	Line bisection task
[200]	Language	Counting Object picture-naming (*DO80*) Nonverbal semantic association test (*PPTT*)
	Language + Motor	Naming + left arm movement
	Face recognition	Famous face naming
	Social cognition	*Reading the Mind in the Eyes Test*
	Visuospatial	Line bisection task
[201]	Language	Picture naming
[202]	Language	Counting Naming Semantic association
	Motor	Hand movement
	Executive function	*Stroop task*
	Praxis	Hand Manipulation Task (*HMt*)
[205]	Language	Free speech Comprehension Counting Picture naming Nonverbal semantic association test (*PPTT*)
[206]	Language	Naming task
	Motor + language	Repeating flexion and contralateral extension + naming
[207]	Language	Counting Object-picture naming (*DO80*)
	Motor + language	Naming / Counting + Continuous movements of contralateral upper extremity
	Motor	Alternating flexion and extension of arm, hand and fingers
[208]	Language	Counting Naming Reading Word and sentence comprehension Repetition tasks
	Calculation	Calculation
[209]	Language	Counting Object naming Single word repetition Syntactic comprehension (2 AFC auditory sentence-to-picture matching task)
[210]	Language	Object naming Repetition of words, pseudowords and phrases Understanding simple and complex orders Verbal fluency
[211]	Language	Counting Picture naming Following commands Reading Naming auditory described objects Following auditory commands
[212]	Language	Object picture-naming (*DO80*) Nonverbal semantic association test (*PPTT*)
	Language + Motor	Naming test + Simple repetitive movements of the contralateral upper limb
	Visuospatial	Line bisection task
[213]	Language	Combination of the *DO80* and a semantically associated verb in the infinitive form
[214]	Praxis	Hand Manipulation Task (*HMt*)
[215]	Language	Naming Semantic association task
	Praxis	Hand Manipulation Task (*HMt*)
	Visuospatial	Visual Field Task
[216]	Praxis	Hand Manipulation task (*HMt*)
[217]	Language	Object picture-naming (*DO80*) Nonverbal semantic association test (*PPTT*)
	Motor	No specific test mentioned
	Social cognition	*Reading the Mind in the Eyes test*
[218]	Language	Naming (*Snodgrass Vanderwaart)*
[219]	Language	Naming task
[220]	Language	Naming Nonverbal semantic association test (*PPTT*)
	Social cognition	Adapted version *Reading the Mind in the Eyes test*
	Visuospatial	Line bisection task
[222]	Motor	Simple hand movements
	Visuospatial	Dot counting
	Left-right orientation	*Bergen Right-Left Discrimination Test*
[223]	Language	Reading
	Motor	Alternately touching thumb to fingers
	Proprioception	Distal phalanx of thumb moved up and down while the experimenter fixed the joint. Patient had to indicate movement direction. * Tested in wrist, elbow, toe, foot and knee
	Calculation	Calculation task
	Clock reading	Clock reading
[224]	Language	Picture naming
	Motor	Flexion and extension of the left arm and hand
	Executive function	*Stroop task*
	Working memory	Digit span Recall
[225]	Language	Picture naming Auditory descriptive naming Non-word repetition Single word reading Writing
	Face recognition	Famous face naming
[226]	Language	Object naming (*Snodgrass naming task)* Reading
	(Working) memory	Digit span forwards Digit span backwards
	Executive functions	DKEFS; *Colour-Word Interference Test*: inhibition condition DKEFS; *Colour-Word Interference Test*: Inhibition/switching condition
	Visuospatial	Dot counting task
[227]	Motor	Verbal commands of voluntary movements
[228]	Motor	Voluntary motor movement based on verbal commands
[229]	Language	Pictured object naming Pronouncing a familiar written Japanese word Action verb generation during image presentation Spontaneous speech through continuous conversation
[230]	Motor	Verbal commands of voluntary movements
[231]	Face recognition	Show faces and ask for change in perception Look at objects and the presenter’s own faces to discriminate face related from object related responses
[232]	Motor	Tapping Continuous movement of the upper limb
[233]	Visuospatial	Detect alteration in visual field using an evaluation chart
[234]	Language	Counting Picture-naming task Verb generation task
	Motor	Bimanual hand-coordination task Finger-to-thumb
[235]	Language	Adapted Boston Naming test Verb generation task Noun generation Counting
[236]	Language	Counting Picture naming Word reading Semantic association task
	Language + Motor	Counting/Picture naming + Contralateral movement
[237]	Motor	Tapping test
	Music	Playing the clarinet
[239]	Language	Counting Picture naming (*DO80*) Nonverbal semantic association test (*PPTT*)
	Language + motor	Counting + contralateral repetitive movement
	Visuospatial	Line bisection task Presentation of 2 pictures diagonally on the screen
	Social cognition	*Reading the Mind in the Eyes Test*
[240]	Language	Counting Picture naming
	Calculation	Calculation task
[241]	Language	Picture naming task
[242]	Language	Counting Days of the week Comprehension tasks Picture descriptions Repetition Reading Writing
[243]	Language	Open conversation Auditory comprehension Naming
[244]	Language	Language switching task: single language naming condition and language-switching condition (Spanish and English)
	Executive function	Language-switching (Spanish and English)
[245]	Language	Simplified version of the naming task (*BNT*) Nonverbal semantic association test (*PPTT*) Semantic pairs test
[246]	Language	Spontaneous speech Object naming
[247]	Motor	Voluntary movements
[248]	Executive function	*Stroop task*
[249]	Motor / Praxis	Hand Manipulation Task (*HMt*) Praxis and motor sequencing Motor planning tasks (*Luria Motor Sequence*)
[250]	Language	Object naming task Verb-generation Action-naming
[251]	Language	Word repetition Sentence repetition
	Motor	Lip pouting Tapping
	Visuospatial	Checkerboard stimulus
[252]	Language	Picture naming task Auditory naming task Answer auditory questions
[253]	Language	Object naming Counting
	Calculation	Calculation
[254]	Language	Counting Naming weekdays, months Object naming Fluency Free dialogue Speech comprehension
[255]	Language	Naming drawn objects
[256]	Language	Counting Naming Spontaneous speech
	Calculation	Calculation
	Visuospatial	Drawing of a dog, in which the dog’s head was in the left superior quadrant and the back legs were in the right inferior quadrant of the visual field. Patient had to indicate when a red laser was visible, which was consecutively pointed to all parts of the picture.
[257]	Language	Picture naming task
[260]	Language	Object naming Sentence reading Speech production Verbal command
	Sensory	Subjective sensory sensations
[261]	Language	Picture-naming task Alternate reading tasks for kana or kanji
[262]	Language	Object naming task
	Visuospatial	Line bisection task
[263]	Language	Word naming tasks (consecutively facial expressions measured during this)
[264]	Language	Counting Picture naming Verb generation Reading kanji and hiragana
	Calculation	Calculation task
[266]	Language	Action verb naming Conceptual knowledge of actions: the Kissing and Dancing Test (*KDT*)
	Sensorimotor	Handedness Decision Task (*HDT*)
	Praxis	Florida Praxis Imagery Questionnaire Buccofacial praxis Ideomotor praxis
	Working memory	Digit span
[267]	Language	Object naming Word reading Word repetition Pseudoword reading Pseudoword repetition Phonological discrimination Verb naming *Token test* Lexical decision
	Working memory	Short term memory span Working memory
[268]	Language	Object naming Verbal fluency Action verb naming Metaphor comprehension
	Executive function	*Stroop task*
	Working memory	Digit span forward Digit span backward
	Processing speed	Symbol Digit Modalities Test (*SDMT*)
[269]	Language	Object naming Phonemic discrimination Word reading Word repetition Pseudoword reading Phonological discrimination Lexical decision and action naming
	Working memory	Digit span
[270]	Language	Counting Reading Repetitive monosyllabic verbalisation
	Motor	Finger tapping task Alternate movements of supination and pronation of the forearm Dorsal and plantar flexion of the ankle *Barré-Mingazzini test*
[271]	Language	Counting Receptive language
[274]	Language	Naming Sentence reading Semantic test of figure association Free dialogue
	Language + Motor	Counting + Flexion and extension of the forearm
	Memory	Memorize a work and repeat it after another image was shown
[275]	Language	Word reading Object naming Visual and nominative semantics Spelling
	Visuospatial	Visual Field Test Drawing Motion cadence
[276]	Praxis	Hand Manipulation Task (*HMt*)
[277]	Language	Counting Object picture naming Action picture naming Word comprehension task Sentence comprehension tasks
	Face recognition	Famous people naming
	Motor	Motor function
	Executive function	*Stroop task*
	Praxis	Hand Manipulation task (*HMt*)
[278]	Language	Standardised naming task Conversation Reading Counting
	Visuospatial	2 pictures in the opposite quadrants
[279]	Language	Visual naming Auditory naming
[280]	Language	Counting Picture naming (*DO80*) Word-reading task
[281]	Language	Word reading task Paragraph reading task Picture naming task Auditory word repetition task Auditory naming task
[282]	Language	Nonverbal semantic association (*PPTT*)
	Language + Motor	Counting + Continuous movement upper limbs
[284]	Language	Counting Picture object naming Reading words from flash cards/screen
	Motor	Stimulation-triggered movement
[286]	Language	Reading Naming objects Auditory naming
[287]	Motor	Execute single brisk wrist extension motion as fast as possible
[288]	Language	Picture naming
[289]	Language	Counting Naming Speech repetition
[290]	Language	Object naming Describing functions of images Answering questions
[291]	Language	Object-picture naming (*DO80*) Nonverbal semantic association (*PPTT*)
	Social cognition	*Reading the Mind in the Eyes Test*
[292]	Language	Naming task (*DO80*) Nonverbal semantic association test (*PPTT*)
	Visuospatial	Line bisection task
	Social cognition	*Reading the Mind in the Eyes Test*
[293]	Language	Counting Naming months Visual naming Auditory naming Repetition
[294]	Language	Counting Object-picture naming (*DO80*)
[296]	Language	Object naming Word repetition
[297]	Language	Counting Reciting a simple Chinese traditional poem Spontaneous speech
[298]	Language	Counting Reciting a simple Chinese traditional poem Free dialogue
[299]	Language	Counting Picture naming Word reading
	Language + Motor	Counting + Alternate flexion and extension of the fingers
[300]	Language	Writing Counting Spontaneous speech Naming Understanding Repetition Fluency
[301]	Language	Counting Object naming Verb generation Nonverbal semantic association (*PPTT*) Reading
	Language + Motor	Counting + Complex motor task (superior and/or inferior limb)
	Visuospatial	Line bisection task Object naming in opposite quadrants
	Social cognition	Modified *Reading the Mind in the eyes Test*
	Executive function	*Stroop task*
[302]	Language	Counting Object naming Nonverbal semantic association test (*PPTT*) Verb generation Reading Comprehension
	Executive function	*Stroop task*
	Social cognition	*Reading the Mind in the Eyes Test*
[303]	Language	Naming actions Finishing sentences Naming objects Phonological discrimination
[1] Video	Language	Counting Naming Reading
	Motor	Alternate hand movement
	Language + Motor	Naming/Reading + Alternate hand movement
	Visuospatial	Line bisection task
[2] Video	Language + Motor	Counting + moving the right arm Object picture-naming (*DO80*) + moving the right arm
[3]	Language + motor	Naming + contralateral motor movement
	Visuospatial	Line bisection task Cancelation task
[4] Video	Language	Object naming Verb generation
[5] Video	Visuospatial	Line bisection task
[6] Video	Language + Motor	Counting + right upper limb movement Naming task + right upper limb movement
[7] Video	Language	Counting Naming Reading
	Motor	Alternative hand movements
	Social cognition	Emotion recognition task
[8] Video	Language	Object naming task
[9]	Language	Naming objects
	Motor	Move right upper extremity

**Table 2:** Overview of the tests or test paradigms that are part of the category “other”

Cognitive domain	Test
Executive functions	Go/No-go task Switch counting and listing the alphabet (1-A-2-B) TMT-B DKEFS; Colour-Word Interference Test: inhibition condition DKEFS; Colour-Word Interference Test: Inhibition/switching condition Stroop Task Language switching
Clock reading	Clock reading test
Face recognition	Famous face naming Show faces and ask for change in perception (distortion of face perception or prosopagnosia)
Musical skills	Tone and rhythm tasks Playing an instrument Listening to music
Proprioception	Distal phalanx of thumb moved up and down while the experimenter fixed the joint. Patient had to indicate movement direction *Tested in wrist, elbow, toe, foot, and knee
Processing speed	Symbol Digit Modalities Test
Left–right orientation	Bergen Right Left Orientation Test

## Discussion

With the shifted focus towards more extensive monitoring of cognition and more person-centered care, we created an overview to see whether the scope of tests used during awake craniotomy has broadened and we present the most important changes over the last years. First and foremost, the language domain continues to be by far the most extensively, and most often, monitored domain during the surgical procedures. It is not surprising that language is such an integral part of almost all awake craniotomies, since this function is highly related to quality of life [82]. Another reason why language is oftentimes monitored is that it relatively easily meets the specific criteria for tests that are used during awake craniotomy which are different than those for the standard neuropsychological tests used in the clinical setting. For example, a stimulus can only be presented for a very short duration because of time of electrical stimulation [191]. Moreover, tests need multiple stimuli with comparable levels of difficulty to allow for repeated measures, but learning effects should remain minimal [221]. To diminish the possibilities of chance-level guessing, multiple choice answers are less desirable. These criteria are easily applicable for language tests, which contributes to the extensive mapping of this domain during surgery. On the other hand, these criteria can explain why other higher-order functions remain underexposed. For instance, memory tasks in general tend to take much more time and raises the question whether stimulation should be applied during the encoding or retrieval phase. These are examples of factors complicating the development of new tests in such cognitive domains. A notable change within the language domain is the increased use of the Pyramid and Palm Trees Test (PPTT; up to 15.4% compared to the previous 2.5%), a test designed to measure nonverbal semantic associations. Recent work shows that there is a dissociation between cortical areas which are associated with verbal semantic cognition and those with nonverbal semantics [99].

Regarding motor and praxis functions, there seems to be an overall percentual increase of studies testing this domain. For praxis, the hand-object manipulation task (HMt), a novel intraoperative task to prevent post-operative apraxia, is reported in 11 included studies (e.g., [75, 76, 202, 214, 216, 277]). The task is useful for testing regions important in motor execution with the dorsal and ventral premotor areas as main stimulation sites impacting different task features. In short, the task consists of a small cylindrical handle which is inserted inside a rectangular base with a worm screw [214]. By means of a precision grip, the patient is sequentially grasping, holding, rotating, and releasing this handle in a self-generated rhythm. Since they receive no external cues, muscle control is solely guided by tactile and proprioceptive information. The task contributes to identification and preservation of dexterous hand movement areas, extending beyond the dorsal premotor areas towards ventral areas within the premotor central gyrus [214]. One of the advantages of this newly developed task is that the rhythmic movement overcomes the problem of the short electrical stimulation criteria and the task minimalizes learning effects. In a case report, praxis and motor sequencing was tested by implementing the Luria Motor Sequence task [249]. Problems with executing this task are associated with kinetic apraxia, which is the inability to correct for erroneous behavior in complex motor sequences [295]. Whereas the authors did not clearly describe how they performed the task during surgery, it is assumed that the underlying principles align with the Hand Manipulation task, since the task concerns sequencing of movements. This would allow for the short periods of electrical stimulation which is necessary in tasks used during awake brain surgery. The importance of bimanual coordination in sports and music has been previously mentioned and it has been noted that patients with frontal glioma can experience permanent deficits in bimanual movements [64, 118]. In the current included studies, there is no clear evidence that this function is tested, but there are six studies that included the finger tapping task, which is often used to study the motor system and can theoretically be used to study bimanual coordination [285].

Notably, compared to the 2% of studies that previously described measuring social cognition, there is currently more attention for this domain as this percentage increased up to 11% [221]. Of this 11%, more than 73% explicitly mention the Reading the Mind in the Eyes Test, which is a well-validated test for face-based mentalizing, that is, the ability to attribute mental states to others [26]. This subserves anticipating the actions of others, but does not involve making inferences about the content or origin of the mental state. Therefore, attribution of the mental state of others based on the area just around the eyes is a part of mentalizing, but is not all of it [26]. The other 27% made use of other tests for social cognition, such as the Pictures of Facial Affect which shows complete faces instead of just the eyes, a false beliefs task measuring theory of mind, or a task designed to predict mental states of others based on a specific arrangement of pictures [140, 175, 176]. The increased use of social cognition monitoring aligns with the preferred shift towards intraoperative mapping of the higher emotional cognitive states in order to avoid long-lasting social cognitive disorders, due to the strong link between preserved social cognition and social interactions [176].

Regarding visuospatial functions, an increase is seen in studies incorporating this domain during mapping, but only a handful of different tests are being used. The importance of monitoring visuospatial deficits subserves preventing post-operative neglect and hemianopia, which both have a highly negative impact on daily functioning [272]. Visual field tests, naming of objects presented diagonally on a screen which is divided in four quadrants, and line bisection tasks are adequate tests to monitor visuospatial functions. An interesting new paradigm that is already incorporated in some studies is the time-to-contact (TTC) test [29]. The task is developed as a measure of time estimation in which an initial part of an object’s trajectory (e.g., a looming ball in a corridor) is presented for a short period of time [33]. Then, the stimulus is shortly occluded and the participant is required to give indication upon the estimated arrival in their peripersonal space. A benefit of this paradigm is that velocity, occlusion time, and trajectory distance can be varied to allow repeated measures while preventing learning. The decision to use the TTC task in current study was to get a more fundamental understanding of the anatomical structures that are involved in TTC estimations [29]. The authors conclude on a role of the right parietal lobe when in the peripersonal space of the observer [29]. However, there is no conclusion yet on whether this network is essential in visuospatial processing in general or only TTC perception. Whereas only preserving TTC perception is interesting for daily life activities such as crossing a street, if the network generalizes to visuospatial processing in general, this specific task will be a more useful addition to the incorporated tests during awake brain surgeries [29, 30]. Therefore, more research is needed in a diverse patient population with visuospatial deficits. As concluded in 2018, we were in specific need of tests in the executive function domain [221]. The only two studies previously included measured inhibition by means of a go-no go task or the Stroop task [221]. Currently, the Stroop task is most often used, but as can be seen in Table 2, there are other tests that can be implemented as well, such as the TMT-B to objectify set-shifting [147]. Another example which we want to highlight is a case study in which shifting between languages is monitored as measure of cognitive control [244].

The famous-face naming task has received increased attention over the past years. This task is particularly important as deficits in naming people is frequently observed in patients with temporal lobe epilepsy [32]. As retrieving proper names by people is a higher order recognition process, the recent focus on assessing higher order cognitive functions might explain the rise of the test [198]. Naming of (famous) faces could also be incorporated to monitor prosopagnosia.

Incorporating digitalized versions of classical neuropsychological tests is a promising approach for awake brain surgery protocols as it offers the possibility to use tests that are difficult to apply as a paper and pencil version. For instance, the conventional TMT cannot be administered effectively due to the brief duration of electrical stimulation and the logistical challenge posed by the lying position of patients during surgery. The use of digitalized versions of tests overcomes this problem as it can provide not only more continuous outcome measures, but also more fine-grained outcome measures such as response time per connected step in the trail instead of solely overall completion time [273]. This can then be used to objectify sustained attention by reaction time measures during one or several tasks every 4 to 5 s [64]. Others used a tablet to measure set shifting by means of the Trail Making Test part B and the digital line bisection task to measure spatial attention [147]. The Symbol Digit Modalities Test to measure processing speed also has a digitalized version that could be incorporated in surgical protocols [195]. Therefore, we hope to see a shift in the upcoming years in which more classical tests will be digitalized to stimulate the use of these during awake craniotomy. Of course, precise, and quantitative registrations from digitalized tests should always go together with more qualitative outcome measures. For example, alterations in the emotional tone of the voice or in patient’s mimic may be an indication of changes in social cognition or emotional expression and may be as relevant as exact response time per item.

The results of this study demonstrate an enormous number of tests or test paradigms that can be used for monitoring different cognitive functions during awake brain surgery. Some of them are frequently used, others still only sporadically. This frequency tells us something about for example the feasibility of the test during surgery. However, the frequency in which a test has previously been used or reported should not be leading in deciding which tasks will be used for an individual patient. For this, other more important factors must be leading, e.g., location of the tumor and the surrounding cortico-subcortical neural circuits, patient’s cognitive complaints, and patient’s wishes [143].

The results of previous review showed that in the majority of studies solely one cognitive domain was monitored during the surgical procedures [221]. In current review, this decreased to 49%, indicating a trend towards monitoring multiple domains and using different tests. Mapping a broader cognitive range can result in more global preservation of cognitive functions. However, not everyone agrees that all (complex) cognitive functions should be monitored during awake surgery. There is an interesting debate about the expansion of cognitive mapping in the context of the onco-functional balance [98, 141]. The fact that complex cognitive functions seem to rely on large-scale networks makes them possibly more difficult to map with electrostimulation [98]. Furthermore, it can be questioned whether neuropsychological tests used during surgery indeed measure the complex cognitive functions you wanted to map [98]. In addition, some cognitive functions are possibly more resilient to damage than others. In contrast, others do advocate developing new tasks to better explore such complex cognitive functions, both extra- and intraoperatively [141]. However, before introducing new tasks that can be used to monitor cognition during awake surgery, their level of evidence should be analyzed in a systematic way [144]. Although the field is quickly developing, many research questions are still in need of being answered. Publishing about cognitive monitoring during awake surgery, specifically about which tests are used to measure what kind of cognitive domains in combination with clear descriptions of outcome measurements (cognitive outcomes, but for example also extent of resection), contributes to best patient care and we therefore recommend these steps for future research.

As with any study, some strengths and limitations should be discussed. An advantage of the method used in current paper is the extensiveness of the search string and that this is an exact replication of previous work so that the results can be compared [221]. Moreover, we have made use of the relatively new artificial tool ASReview, which has been proven to be efficient and reliable [238]. However, using machine learning–based screening system does have drawbacks. For example, the tool does not provide an accurate estimation of the system’s error rate and bias in data extraction and coding remains present [238]. That being said, screening by humans remains imperfect and mistakes can have been made during the labeling of studies [283]. Furthermore, with the large number of included studies in this review, we do not expect that the results would deviate a lot from current findings depending on missed articles or wrongfully excluded articles (due to human error).

## Conclusion

In conclusion, the current study indicates that there is a positive trend towards implementation of a broader range of tests during awake brain surgery. We see a shift towards more extensive monitoring during the procedures, especially in the domains of motor functioning, social cognition, visuospatial processing, and executive functioning. In order to achieve more extensive cognitive monitoring, implementations of new tests, revised tests, or digital versions of more traditional neuropsychological tests during surgery offer opportunities for the future. We hope to see that this process will be continued during the upcoming years to increase the quality of life after awake craniotomy and to strengthen the focus on the specific needs of the patients.

## Supplementary Information

Below is the link to the electronic supplementary material.

Supplementary file1 (DOCX 13 KB)

## Data Availability

Data is available upon request.

## Abbreviations

DO80 Object picture-naming test: Test de Dénomination Orale D’Image
PPTT: Pyramid and Palm Trees Test
AI: Artificial Intelligence
PRISMA: Preferred Reporting Items for Systematic Reviews and Meta-Analysis
TMT: Trail Making Test
TTC: Time-To-Contact
HMT: Hand-object Manipulation Task
SDMT: Symbol Digit Modalities Test
