# Supplementary material for: An update on tests used for intraoperative monitoring of cognition during awake craniotomy
Source: Acta Neurochir (Wien). 2024 May 7;166(1):204. doi: 10.1007/s00701-024-06062-6 (PMC11076349; doi:10.1007/s00701-024-06062-6)
Supplement: Supplementary file 1 — Supplementary file1 (DOCX 13 KB) [file 701_2024_6062_MOESM1_ESM.docx]

**Supplementary**

Search strategy used for Pubmed and Embase

*Pubmed*. ("Brain Neoplasms"[MeSH:noexp] OR brain neoplasm*[Title/ Abstract] OR brain tumor*[Title/Abstract] OR brain tumour* [Title/Abstract] OR "Glioma"[Mesh] OR glioma*[Title/Abstract] OR glioblastoma*[Title/Abstract] OR "Epilepsy"[MAJR] OR epilepsy[Title/Abstract] OR epilepsies[Title/Abstract]) AND ("Neurosurgical Procedures"[Mesh] OR "Cytoreduction Surgical Procedures"[Mesh] OR Cytoreduction Surgical Procedure*[Title/Abstract] OR reduction[Title/Abstract] OR debulking[Title/Abstract] OR remove[Title/Abstract] OR removal[Title/Abstract] OR surgery[Title/Abstract] OR craniotomy[Title/Abstract]) AND ("Anesthesia, Local"[Mesh] OR local anesthesia[Title/Abstract] OR local anaesthesia[Title/Abstract] OR "Transcranial Direct Current Stimulation"[Mesh] OR stimulat*[Title/Abstract] OR awake[Title/Abstract] OR penfield[Title/ Abstract] OR "Monitoring, Intraoperative"[Mesh] OR intraoperative monitoring[Title/Abstract]).

Filters applied: from 2017/3/1 – 2023/11/10

*Embase* : 'brain tumor'/exp OR 'brain tumor' OR 'brain'/exp OR brain) AND neoplasm*:ti,ab,kw OR 'brain'/exp OR brain) AND tumour*:ti,ab,kw OR 'glioma'/exp OR 'glioma' OR glioma*:ti,ab,kw OR glioblastoma*:ti,ab,kw OR 'epilepsy'/mj/exp OR 'epilepsy' OR 'epilepsy':ti,ab,kw OR epilepsies:ti,ab,kw) AND ('cytoreductive surgery'/exp OR 'cytoreductive surgery') OR 'neurosurgery'/exp OR 'neurosurgery' OR cytoreductive) AND surgery:ti,ab,kw OR reduction:ti,ab,kw OR debulking:ti,ab,kw OR remove:ti,ab,kw OR removal:ti,ab,kw OR surgery:ti,ab,kw OR craniotomy:ti,ab,kw) AND ('local anesthesia'/exp OR 'local anesthesia') OR 'local anaesthesia':ti,ab,kw OR 'transcranial direct current stimulation'/exp OR 'transcranial direct current stimulation' OR stimulat*:ti,ab,kw OR awake:ti,ab,kw OR penfield:ti,ab,kw OR 'neuromonitoring'/exp OR 'neuromonitoring' OR intraoperative) AND monitoring:ti,ab,kw AND [embase]/lim NOT [medline]/lim AND ('article'/it OR 'article in press'/it OR 'review'/it) AND [english]/lim

Publication years from 2017 – 2023. Searched on 2023/11/10
